# Supplementary material for: Adiposity and risk of prostate cancer death: a prospective analysis in UK Biobank and meta-analysis of published studies
Source: BMC Med. 2022 May 5;20:143. doi: 10.1186/s12916-022-02336-x (PMC9069769; doi:10.1186/s12916-022-02336-x)
Supplement: Supplementary file 1 — Additional file 1: Supplementary Methods. Meta-analyses from prospective studies, literature search, study selection, data extraction, displaying of findings. Figure S1. Flow chart of the study participants in UK Biobank. Figure S2. Flow diagram of literature search and study selection for the meta-analysis. Table S1. Characteristics of prospective studies and previous individual participant data meta-analysis of body mass index and prostate cancer death. Table S2. Characteristics of prospective studies and previous individual participant data meta-analysis of body fat percentage, waist circumference and prostate cancer death. Table S3. Characteristics of prospective studies and previous individual participant data meta-analysis of waist to hip ratio and prostate cancer death. Table S4. Baseline characteristics of participants according to fourths of BMI at recruitment in men from UK Biobank. Table S5. Baseline characteristics of participants according to fourths of waist at recruitment in men from UK Biobank. Table S6. Mean and SD in men from UK Biobank with available imaging data (up to 4800 men). Table S7. Pearson correlation coefficients between main adiposity measurements at baseline in 218,237 men from UK Biobank. Table S8. Pearson correlation coefficients between adiposity measurements (imaging visit) with MRI adiposity measurements from the imagining in up to 11,501 men from UK Biobank. Table S9. Pearson correlation coefficients between adiposity measurements (imaging visit) with DXA adiposity measurements from the imagining in up to 18,827 men from UK Biobank. Table S10. Mean difference in MRI- and DXA-derived body fat compartments per 1 SD higher levels of BMI, body fat percentage, waist circumference, and waist to hip ratio in men from UK Biobank. Table S11. Geometric means of selected MRI measurements by tenths of anthropometric measurements at the imaging visit in up to 11,501 men from UK Biobank. Table S12. Geometric means of selected DXA measurements [file 12916_2022_2336_MOESM1_ESM.docx]

**Additional file 1: Adiposity and risk of prostate cancer death: a prospective analysis in UK Biobank and meta-analysis of published studies**

[**Additional File 1: Supplementary Methods** 3](#_Toc97825089)

[Meta-analyses from prospective studies 3](#_Toc97825090)

[Literature search 3](#_Toc97825091)

[Study selection 3](#_Toc97825092)

[Data extraction 4](#_Toc97825093)

[Displaying of findings 4](#_Toc97825094)

[**Additional File 1: Figures** 5](#_Toc97825095)

[**Figure S1.** Flow chart of the study participants in UK Biobank. 5](#_Toc97825096)

[**Figure S2.** Flow diagram of literature search and study selection for the meta-analysis. 6](#_Toc97825097)

[**Additional File 1: Tables** 8](#_Toc97825098)

[**Table S1.** Characteristics of prospective studies and previous individual participant data meta-analysis of body mass index and prostate cancer death. 8](#_Toc97825099)

[**Table S2**. Characteristics of prospective studies and previous individual participant data meta-analysis of body fat percentage, waist circumference and prostate cancer death. 12](#_Toc97825100)

[**Table S3**. Characteristics of prospective studies and previous individual participant data meta-analysis of waist to hip ratio and prostate cancer death. 14](#_Toc97825101)

[**Table S4.** Baseline characteristics of participants according to fourths of BMI at recruitment in men from UK Biobank. 15](#_Toc97825102)

[**Table S5.** Baseline characteristics of participants according to fourths of waist at recruitment in men from UK Biobank. 16](#_Toc97825103)

[**Table S6:** Mean and SD in men from UK Biobank with available imaging data (up to 4,800 men). 17](#_Toc97825104)

[**Table S7.** Pearson correlation coefficients between main adiposity measurements at baseline in 218,237 men from UK Biobank. 18](#_Toc97825105)

[**Table S8.** Pearson correlation coefficients between adiposity measurements (**imaging visit**) with MRI adiposity measurements from the imagining in up to 11,501 men from UK Biobank. 19](#_Toc97825106)

[**Table S9.** Pearson correlation coefficients between adiposity measurements (imaging visit) with DXA adiposity measurements from the imagining in up to 18,827 men from UK Biobank. 20](#_Toc97825107)

[**Table S10**. Mean difference in MRI- and DXA-derived body fat compartments per 1 SD higher levels of BMI, body fat percentage, waist circumference, and waist to hip ratio in men from UK Biobank. 21](#_Toc97825108)

[**Table S11**. Geometric means of selected MRI measurements by tenths of anthropometric measurements at the imaging visit in up to 11,501 men from UK Biobank. 22](#_Toc97825109)

[**Table S12**. Geometric means of selected DXA measurements by tenths of anthropometric measurements **at the imaging visit** in up to 18,827 men from UK Biobank. 24](#_Toc97825110)

[**Table S13.** Minimally- and multivariable-adjusted hazard ratios (95% CI) for prostate cancer death in relation to adiposity measurements at baseline in men from UK Biobank. 26](#_Toc97825111)

[**Table S14.** Multivariable-adjusted hazard ratios (95 % CI) for prostate cancer in relation to BMI, waist circumference and WHR using the WHO cut-off points at recruitment in men from UK Biobank. 27](#_Toc97825112)

[References 28](#_Toc97825113)

# Additional File 1: Supplementary Methods

## **Meta-analyses from prospective studies**

The latest World Cancer Research Fund (WCRF) meta-analysis did not have enough data from previous prospective studies to look at the association of central adiposity measurements (i.e. waist circumference and waist to hip ratio (WHR)) with prostate cancer mortality ^1^. Moreover, a recent pooled analysis from prospective studies found a positive association of both total and central adiposity with prostate cancer mortality, but this study included 22% of all the worldwide prospective data for total adiposity and 50% for central adiposity ^2^.

To put our findings in UK Biobank into the context of previous research, we conducted an updated meta-analysis combining our results with those from previously published prospective studies of the association between total and central adiposity and prostate cancer death, following standard criteria for meta-analyses (the MOOSE guidelines) ^3^.

### Literature search

We searched on PubMed, Embase, and Web of Science for prospective studies examining the association of BMI, waist circumference and WHR with prostate cancer as the underlying cause of death independently by two researchers up to 15^th^ March 2021. We used the search terms “obesity”, “adiposity”, “body mass index”, “body mass”, “waist circumference”, “waist hip ratio”, “anthropometry”, “body composition”, “body size”, “body fat”, “prospective”, “cohort”, “prostate”, “cancer”, “carcinoma”, “tumour”, “neoplasm”, “death”, “mortality”, “fatal”, and “lethal”. After the removal of duplicate studies, titles and abstracts were independently screened by the two trained researchers using the Rayyan QRCI web application (<https://rayyan.qcri.org/>) ^4^.

### Study selection

The inclusion criteria for the studies were: 1) prospective cohort studies; 2) studies that investigated the associations between BMI, body fat, waist circumference and/or WHR and prostate cancer as the underlying cause of death; 3) studies reporting hazard ratio (HR) or relative risk (RR) with the corresponding measure of variability [95% confidence intervals (CI)].

When the same cohort study published more than one original articles looking at these associations, the paper reporting the longest follow-up time was kept. Since the previous pooled analysis of individual participant data from prospective studies did not report risk of prostate cancer mortality separately in the individual studies and most of these studies have not published an original article looking at adiposity measurements and prostate cancer death, we included the pooled estimate published in the pooling project ^2^, and those studies included in this pooling project were not separately included in the meta-analysis (Supplementary Figure 2).

### Data extraction

For each study we extracted the fully adjusted HRs and their 95% CIs of the lineal (per increment) association between the adiposity measurement and prostate cancer mortality. If comparison per increment was not available we extracted the categorical comparison. For all studies, only results with the most comprehensive adjustment for confounders were considered.

Supplementary tables 1-3 show the name of the first author, publication year, country or region, age at recruitment, years of follow-up, sample size and number of prostate cancer deaths, confounder adjustments used in each study, comparison made, HR estimates and their corresponding 95% CI, and conversion of HR and 95% CI to per increment if per increment results were not reported in the original study.

### Displaying of findings

In order to include all prospective analyses on the same continuous scale (e.g. per 5 kg/m^2^ increase in BMI), we estimated these effect measures from the available data reported in each study as follows:

- Studies that reported associations in the same continuous scale that the one we want to use in our continuous meta-analysis: This continuous scale was used in our meta-analysis.
- Studies that reported associations in a different continuous scale that the one we want to use in our continuous meta-analysis: HR and 95% CI were rescaled to the change used in this meta-analyses by 𝐻𝑅_𝑦_=(𝐻𝑅_𝑥_)^𝑦/𝑥^, where the HR for an increase in 𝑦 units of the predictor variable (𝐻𝑅_𝑦_) is equal to the HR for an increase in 𝑥 units (𝐻𝑅_𝑥_) raised to the power of 𝑦/𝑥; the sample principle applies for the upper and lower 95% CI.
- Studies that reported categorical associations instead of associations in a continuous scale:

For all studies, an effect estimate and its standard error (SE) on the same scale (e.g. per 5 kg/m^2^ increase in BMI) are necessary for inclusion in a ‘continuous’ meta-analysis. For BMI-prostate cancer studies, the effect estimate could be hazard ratio (HR) and risk ratio (RR). Summary relative risks combining study-specific results were estimated by calculating the weighted average of the study-specific logarithms of the relative risks, with weights proportional to the inverses of the variances of the study-specific log relative risks. (NB: calculation of such a weighted average is sometimes referred to as a fixed-effect meta-analysis). Chi-squared tests were used to assess heterogeneity across studies.

# Additional File 1: Figures


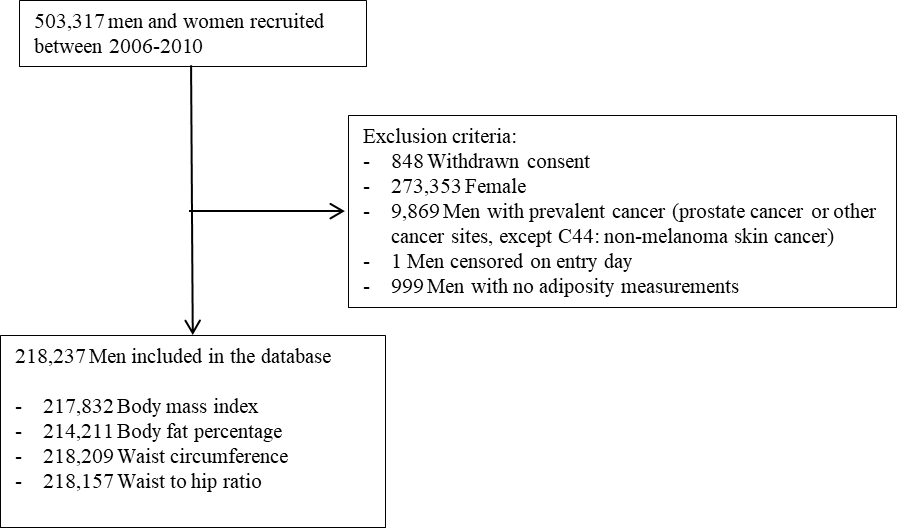


## **Figure S1.** Flow chart of the study participants in UK Biobank.


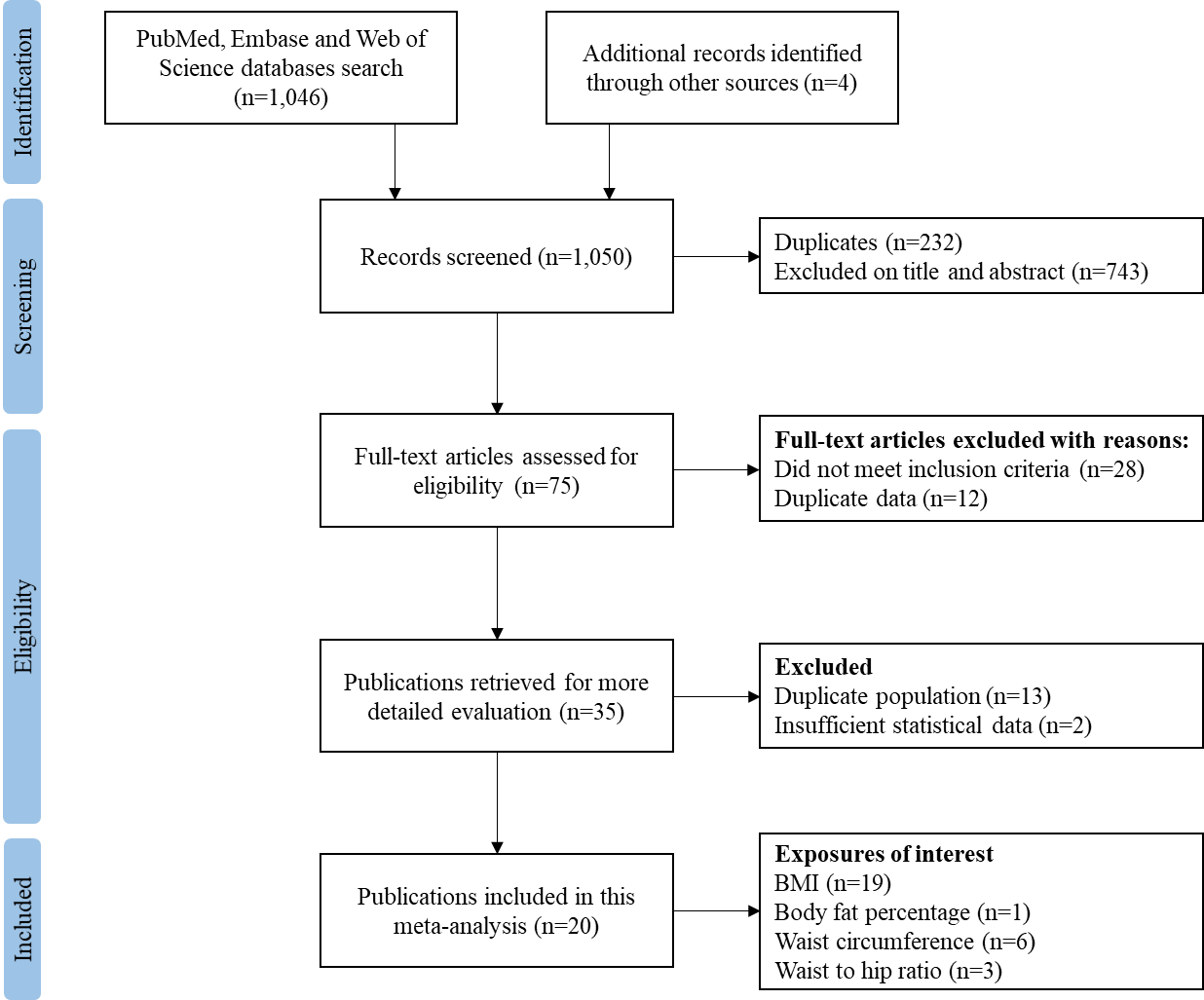


## **Figure S2.** Flow diagram of literature search and study selection for the meta-analysis.

A)


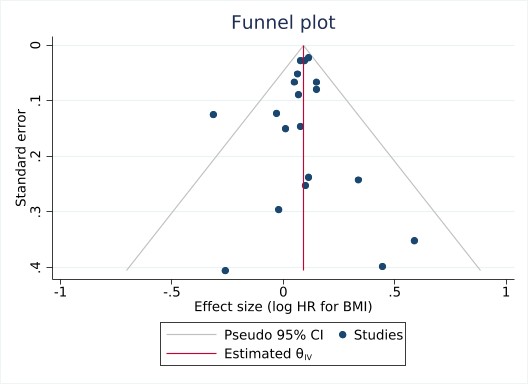


B)


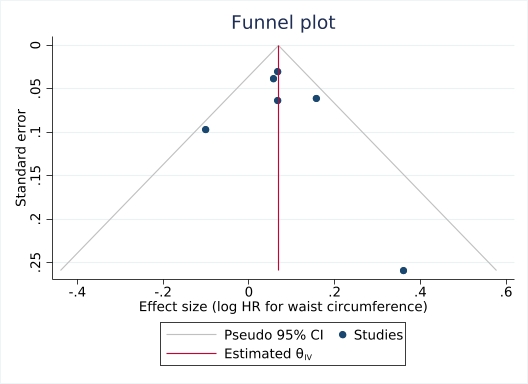


**Figure S3.** Funnel plot of studies reporting (A) BMI and (B) waist circumference.

# Additional File 1: Tables

| Table S1. Characteristics of prospective studies and previous individual participant data meta-analysis of body mass index and prostate cancer death. | | | | | | | | |
| --- | --- | --- | --- | --- | --- | --- | --- | --- |
| Authors, Year (Reference) | **Study** | **Country (Age, follow-up)** | **Pca death/total sample** | **Exposure categories** | **HR** | **95% CI** | **Covariates** | **Conversion of HR (95% CI) to per 5 kg/m^2^ increment ^1^** |
| Gapstur et al., 2001 ^5^ | The Chicago Heart  Association  (CHAC) | USA  (Mean age at baseline: 40y  Mean follow-up: 27y) | 176/20,433 | Per 1 SD increase (SD=4 kg/m^2^) | 0.98 | 0.81-1.12 | Age, plasma glucose concentrations, heart rate, education, race | 0.97 (0.77-1.15) |
| Rodriguez et al., 2001 ^6^ | Cancer  Prevention  Study I  Nutrition  Cohort Study (CPS I) | USA  (Median age at baseline: 52y  Mean follow-up: 13y) | 1590/456,490 | ≥32.50 kg/m^2^ *vs* 18.50–22.49 kg/m^2^ | 1.34 | 0.93-1.94 | Age at interview, race, height, education, exercise, smoking status, and family history of prostate cancer | 1.05 (0.93-1.19) |
| Calle et al., 2003 ^7^ | Cancer  Prevention  Study II  Nutrition  Cohort Study (CPS II) | USA  (Mean age at baseline: 57y  Mean follow-up: 16y) | 4004/404,576 | ≥35 vs. < 25  kg/m^2^ | 1.34 | 0.98-1.83 | Age, education, smoking status and number of cigarettes smoked, physical activity, alcohol use, marital status, race, aspirin use, fat consumption, and vegetable consumption | 1.08 (1.02-1.13) |
| Eichholzera et al., 2005 ^8^ | Basel Prospective Study | Switzerland (Mean age at baseline: not available  Mean follow-up: 17y) | 30/2974 | Per unit increase | 0.95 | 0.93-1.18 | Smoking status and age group | 0.77 (0.43-1.40) (final estimate taken from WCRF meta-analysis as the lower CI didn’t look correct in the original publication |
| Fujino et al., 2007 ^9^ | Japan  Collaborative  Cohort Study (JACC) | Japan  (Mean age at baseline: not available  Mean follow-up: ~5y (exact follow-up not given)) | 156/~110,700 (exact total n not included in original publication | ≥30 vs. 18.5-24.9 kg/m^2^ | 0.87 | 0.12-6.29 | Age and area of study | 1.40 (1.00-1.95) |
| Martin et al., 2009 ^10^ | Nord-Trøndelag  Health Study  (HUNT 2) | Norway (Mean age at baseline: 48y  Mean follow-up: 9.3y) | 110/29,364 | Per 1 SD increase, 3.5 kg/m^2^ | 1.01 | 0.83-1.24 | Age, height, smoking, marital status, education, physical activity, International Prostate Symptom Score | 1.01 (0.77-1.36) |
| Burton et al., 2010 ^11^ | The Glasgow Alumni Cohort | UK (Mean age at baseline: 20y  Mean follow-up: 49y) | 102/9,549 | Per 1 kg/m^2^ increase | 1.02 | 0.93-1.11 | Smoking, father’s social class, and height | 1.10 (0.70-1.68) |
| Batty et al., 2011 ^12^ | Whitehall study (WS) | UK (Mean age at baseline: ~50y (exact age not given)  Mean follow-up: 40y) | 578/17,934 | Per 1 SD increase, 2.96 kg/m^2^ | 1.04 | 0.94-1.14 | Plasma cholesterol, physical activity, socio-economic status, diabetes/blood glucose, marital status, pulmonary function, height, age at risk, smoking, and diastolic and systolic blood pressure | 1.07 (0.90-1.25) |
| Dehal et al., 2011 ^13^ | Nutrition  Examination Survey  Epidemiology  Follow-Up  Study (NHEFS) | USA (Mean age at baseline: 47y  Mean follow-up: 17y) | 44/3,127 | ≥30 vs. 18.5-24.9 kg/m^2^ | 1.36 | 0.53-3.47 | Race, education attainment, family income level, marital status, types of residence area assessed at the baseline, alcohol drinking, cigarette smoking, and frequency of eating fruit and vegetables | 1.12 (0.75-1.68) |
| Discacciati et al., 2011 ^14^ | Swedish men cohort | Sweden (age at baseline: aged 45–79y  Mean follow-up: 9y) | 225/36,959 | Per 5 km/m^2^ increase | 0.73 | 0.53-1.02 | BMI at age 30 years, age at baseline, total energy intake, total physical activity, years of education, smoking status, family history of prostate cancer and personal history of diabetes | 0.73 (0.53-1.02) |
| Gray et al., 2012 ^15^ | Harvard Alumni Health Study | USA (Mean age at baseline: 18.4y  Mean follow-up: 56.5y) | 417/19,593 | 2.56 kg/m^2^ | 1.04 | 0.91–1.19 | Age, cigarette smoking status and physical activity at college entry and BMI in 1962/1966 | 1.08 (0.83-1.40) |
| Haggstrom et al., 2012 ^16^ | Metabolic Syndrome and Cancer Project | USA (Mean age at baseline: 44y  Mean follow-up: 12y) | 961/289,866 | Quintiles, Q5 *vs* Q1 | 1.36 | 1.08-1.71 | Smoking and quintiles of BMI (except for BMI) and stratified for subcohort, 5 birth cohorts, and 5 categories of age at measurement. | 1.16 (1.03-1.29) |
| Taghizadeh et al., 2015 ^17^ | Vlagtwedde-Vlaardingen cohort study | The Netherlands (Mean age at baseline: 45.9y  Mean follow-up: 15.5y) | 61/3718 | ≥30 vs. 18.5-24.9 kg/m^2^ | 3.33 | 1.31-8.46 | Age, smoking habits, and place of residence | 1.8 (1.21-2.59) |
| Hong et al., 2016 ^18^ | Korean Veterans Health Study | Korean (Mean age at baseline: 58.9y  Mean follow-up: 6.4y) | 46/113,478 | Per 5 kg/m^2^ increase | 0.98 | 0.56-1.72 | Age at baseline, smoking status, alcohol consumption, monthly household income, and physical activity | 0.98 (0.56-1.72) |
| Perez-Cornago et al., 2017 ^19^ | EPIC (not included in DCPP, follow-up from 2004-2010) | Europe (Mean age at baseline: 52y  Mean follow-up: 6y) | 670/129,008 | Per 5 kg/m^2^ | 1.16 | 1.02-1.33 | Age, education level, smoking status, marital status, diabetes, and physical activity | 1.16 (1.02-1.33) |
| Dickerman et al., 2019 ^20^ | Age, Gene/Environment Susceptibility–Reykjavik (AGES-Reykjavik) study | Iceland (Mean age at baseline: not available  Mean follow-up: 10.4y) | 31/1832 | Per 5 kg/m^2^ increase | 1.56 | 0.97-2.53 | Age at study entry, family history of prostate cancer, smoking status, education, physical activity, and physician visit over the past 12 months | 1.56 (0.97-2.53) |
| Jochems et al., 2020 ^21^ | Five Swedish cohorts pooling project | Sweden (Mean age at baseline: 37.5y  Mean follow-up: 28y) | 6748/431,902  (170 deaths from MDCS and 54 deaths from VIP likely to also be included in EPIC above) | Per 5 kg/m^2^ increase | 1.12 | 1.08-1.17 | Stratified by cohort and birth period, and adjusted for baseline age, baseline smoking status, healthcare region, country of birth and education | 1.12 (1.08-1.17) |
| Genkinger et al., 2020 ^2^ | Diet and Cancer Pooling Project (DCPP) ^2^ | World-wide pooling project (Mean age at baseline: not available  Mean follow-up: not available) | 3027/830,772  (279 deaths from CPS-II likely to also be included in the separate CPS-II above) | Per 5 kg/m^2^ | 1.10 | 1.05-1.16 | Age, year of questionnaire return, race, education, marital status, alcohol, smoking habits, height, physical activity, prostate cancer family history, personal history of diabetes, multiple vitamin use, dietary calcium, dietary lycopene, and total energy intake | 1.10 (1.05-1.16) |
| Perez-Cornago et al., [this report] | UK Biobank | UK (Mean age at baseline: 56.5y  Mean follow-up: 10.9y) | 571/217,841 | Per 5 kg/m^2^ | 1.08 | 0.97-1.19 | Region, age at recruitment, Townsend deprivation score, ethnicity, lives with a wife or partner, smoking, physical activity, alcohol consumption, height, and diabetes | 1.08 (0.97-1.19) |
| ^1^ Conversion explained in the Supplementary methods  ^2^ Studies included: ATBC, Alpha-Tocopherol Beta-Carotene Cancer Prevention Study; CPS II, Cancer Prevention Study II Nutrition Cohort; COSM, Cohort of Swedish Men; EPIC European Prospective Investigation into Cancer and Nutrition (follow-up only from baseline to 2004); HPFS, Health Professionals Follow-up Study; MCCS, Melbourne Collaborative Cohort Study; MDCS, Malmö Diet and Cancer Study; MEC, Multiethnic Cohort; NLCS, Netherlands Cohort Study; NIH-AARP, The NIH-AARP Diet and Health Study; and PLCO, Prostate, Lung, Colorectal, Ovarian Cancer Screening Trial; VIP, Västerbotten Intervention Project.  Abbreviations: BMI, body mass index; CI, confidence interval; HR, hazard ratio; Pca, prostate cancer. | | | | | | | | |

| **Table S2**. Characteristics of prospective studies and previous individual participant data meta-analysis of body fat percentage, waist circumference and prostate cancer death. | | | | | | | | |
| --- | --- | --- | --- | --- | --- | --- | --- | --- |
| **Authors, Year (Reference)** | **Study** | **Country (Age, follow-up)** | **Pca death/total sample** | **Exposure categories** | **HR** | **95% CI** | **Covariates** | **Conversion of HR (95% CI) to per 5 % increment** ^1^ |
| **Body fat percentage** |  |  |  |  |  |  |  |  |
| Dickerman et al., 2019 ^20^ | Age, Gene/Environment Susceptibility–Reykjavik (AGES-Reykjavik) study | Iceland (Mean age at baseline: not available  Mean follow-up: 10.4y) | 25/1425 | Per 1 SD increase, 5.3 % | 1.20 | 0.80-1.81 | Age at study entry, family history of prostate cancer, smoking status, education, physical activity, and physician visit over the past 12 months | 1.19 (0.81-1.75) |
|  |  |  |  |  |  |  |  |  |
| **Waist circumference** |  |  |  |  |  |  |  | **Conversion of HR (95% CI) to per 10 cm increment** ^1^ |
| Martin et al., 2009 ^10^ | Nord-Trøndelag  Health Study  (HUNT 2) | Norway (Mean age at baseline: 48y  Mean follow-up: 9.3y) | 107/29,364 | Per 1 SD increase, 9.4 cm | 0.91 | 0.74-1.12 | Age, height, smoking, marital status, education, physical activity, International Prostate Symptom Score | 0.90 (0.74-1.12) |
| Perez-Cornago et al., 2017 ^19^ | EPIC (not included in DCPP, follow-up from 2004-2010) | Europe (Mean age at baseline: 52y  Mean follow-up: 6y) | 632/129,008 | Per 10 cm increase | 1.17 | 1.06-1.30 | Age, education level, smoking status, marital status, diabetes, and physical activity | 1.17 (1.06-1.30) |
| Dickerman et al., 2019 ^20^ | Age, Gene/Environment Susceptibility–Reykjavik (AGES-Reykjavik) study | Iceland (Mean age at baseline: not available  Mean follow-up: 10.4y) | 31/1832 | Per 1 SD increase, 10.3 cm | 1.45 | 1.01 -2.07 | Age at study entry, family history of prostate cancer, smoking status, education, physical activity, and physician visit over the past 12 months | 1.43 (1.01-2.03) |
| Genkinger et al., 2020 ^2^ | Diet and Cancer Pooling Project (DCPP) | World-wide pooling project (Mean age at baseline: not available  Mean follow-up: not available) | 1365/586,361 | Per 10 cm increase | 1.07 | 1.01-1.13 | Age, year of questionnaire return, race, education, marital status, alcohol, smoking habits, height, physical activity, prostate cancer family history, personal history of diabetes, multiple vitamin use, dietary calcium, dietary lycopene, and total energy intake | 1.07 (1.01-1.13) |
| Jochems et al., 2021 ^22^ | Five Swedish cohorts pooling project | Sweden (Mean age at baseline: 37.5y  Mean follow-up: 28y) | 387/58,457  (170 deaths from MDCS and 54 deaths from VIP likely to also be included in EPIC) | Per 10 cm increase | 1.07 | 0.95-1.20 | Stratified for cohort and birth period, and adjustment for age at study enrolment, smoking status at study enrolment, healthcare region, country of birth, highest education at study enrolment, and height | 1.07 (0.95-1.20) |
| Perez-Cornago et al., [this report] | UK Biobank | UK (Mean age at baseline: 56.5y  Mean follow-up: 10.9y) | 571/218,218 | Per 10 cm increase | 1.09 | 1.01-1.17 | Region, age at recruitment, Townsend deprivation score, ethnicity, lives with a wife or partner, smoking, physical activity, alcohol consumption, height, and diabetes | 1.09 (1.01-1.17) |
| ^1^ Conversion explained in the Supplementary methods.  ^2^ Studies included: CPS II, Cancer Prevention Study II Nutrition Cohort; COSM, Cohort of Swedish Men; EPIC European Prospective Investigation into Cancer and Nutrition (follow-up only from baseline to 2004); HPFS, Health Professionals Follow-up Study; MCCS, Melbourne Collaborative Cohort Study; **MDCS,** Malmö Diet and Cancer Study; NIH-AARP, The NIH-AARP Diet and Health Study; VIP, Västerbotten Intervention Project**.**  Abbreviations: CI, confidence interval; HR, hazard ratio; Pca, prostate cancer. | | | | | | | | |

| **Table S3**. Characteristics of prospective studies and previous individual participant data meta-analysis of waist to hip ratio and prostate cancer death. | | | | | | | | |
| --- | --- | --- | --- | --- | --- | --- | --- | --- |
| **Authors, Year (Reference)** | **Study** | **Country (Age, follow-up)** | **Pca death/total sample** | **Exposure categories** | **HR** | **95% CI** | **Covariates** | **Conversion of HR (95%) to per 0.05** **increment** ^1^ |
| Martin et al., 2009 ^10^ | Nord-Trøndelag  Health Study  (HUNT 2) | Norway (Mean age at baseline: 48y  Mean follow-up: 9.3y) | 110/29,364 | Per 1 SD increase, 0.06 unit | 0.91 | 0.75-1.11 | Age, height, smoking, marital status, education, physical activity, International Prostate Symptom Score | 0.92 (0.79-1.09) |
| Perez-Cornago et al., 2017 ^19^ | European Prospective Investigation into Cancer and Nutrition (EPIC) | Europe (Mean age at baseline: 52y  Mean follow-up: 6y) | 870/141,896 | Per 0.1 unit increment | 1.18 | 1.08 - 1.28 | Age, education level, smoking status, marital status, diabetes, and physical activity | 1.07 (1.00-1.14) |
| Perez-Cornago et al., [this report] | UK Biobank | UK (Mean age at baseline: 56.5y  Mean follow-up: 10.9y) | 521340 | Per 0.05 unit increment | 1.10 | 1.03-1.17 | Region, age at recruitment, Townsend deprivation score, ethnicity, lives with a wife or partner, smoking, physical activity, alcohol consumption, height, and diabetes | 1.10 (1.03-1.17) |
| ^1^ Conversion explained in the Supplementary methods.  Abbreviations: CI, confidence interval; HR, hazard ratio; Pca, prostate cancer. | | | | | | | | |

| **Table S4.** Baseline characteristics of participants according to fourths of BMI at recruitment in men from UK Biobank. | | | | |
| --- | --- | --- | --- | --- |
|  | **BMI** | | | |
| **Characteristic** | **Q1** | **Q2** | **Q3** | **Q4** |
| No. of men | 55665 | 54194 | 54692 | 53281 |
|  |  |  |  |  |
| Sociodemographic |  |  |  |  |
| Age at recruitment (years), mean (SD) | 56.0 (8.4) | 56.7 (8.3) | 56.8 (8.2) | 56.7 (8.0) |
| Most deprived quintile, % (n) | 11643 (20.9) | 9669 (17.8) | 10448 (19.1) | 12876 (24.2) |
| No qualifications, % (n) | 6738 (12.1) | 7160 (13.2) | 7699 (14.1) | 7805 (14.6) |
| Black ethnicity, % (n) | 666 (1.2) | 744 (1.4) | 893 (1.6) | 915 (1.7) |
| Not in paid/self-employment, % (n) | 21148 (38.0) | 20529 (37.9) | 21022 (38.4) | 21592 (40.5) |
| Living with partner, % (n) | 40889 (73.5) | 42618 (78.6) | 43044 (78.7) | 39610 (74.3) |
|  |  |  |  |  |
| Anthropometric |  |  |  |  |
| Height (cm), mean (SD) | 176.2 (7.0) | 175.8 (6.8) | 175.5 (6.8) | 175.1 (6.8) |
| BMI (kg/m^2^), mean (SD) | 23.2 (1.5) | 26.2 (0.7) | 28.6 (0.8) | 33.6 (3.4) |
| Body fat (%), mean (SD) | 19.5 (4.5) | 23.8 (3.6) | 26.6 (3.4) | 31.4 (4.1) |
| Waist circumference (cm), mean (SD) | 85.8 (6.3) | 93.1 (5.4) | 99.0 (5.7) | 110.4 (9.6) |
| Waist to hip ratio, mean (SD) | 0.886 (0.054) | 0.922 (0.050) | 0.948 (0.051) | 0.988 (0.059) |
|  |  |  |  |  |
| Lifestyle |  |  |  |  |
| Current cigarette smokers, % (n) | 8453 (15.2) | 6348 (11.7) | 6316 (11.5) | 6040 (11.3) |
| Drinking alcohol ≥ 20 g/day, % (n) | 21115 (37.9) | 24016 (44.3) | 25367 (46.4) | 23790 (44.7) |
| Physically inactive, % (n) | 12975 (23.3) | 13380 (24.7) | 15124 (27.7) | 18534 (34.8) |
|  |  |  |  |  |
| Health status |  |  |  |  |
| Vasectomy, % (n) | 2594 (4.7) | 2876 (5.3) | 3087 (5.6) | 2779 (5.2) |
| Hypertension, % (n) | 22203 (39.9) | 27265 (50.3) | 31021 (56.7) | 33155 (62.2) |
| Diabetes, % (n) | 1655 (3.0) | 2239 (4.1) | 3608 (6.6) | 7503 (14.1) |
|  |  |  |  |  |
| Prostate specific factors prior recruitment |  |  |  |  |
| PSA test before baseline, % (n) | 15252 (27.4) | 15731 (29.0) | 15465 (28.3) | 13896 (26.1) |
| Enlarged prostate, % (n) | 1803 (3.2) | 1896 (3.5) | 1805 (3.3) | 1558 (2.9) |
| Family history of prostate cancer, % (n) | 4208 (7.6) | 4159 (7.7) | 4121 (7.5) | 3868 (7.3) |
| ^1^ Values are means (SD).  Abbreviations: BMI, body mass index; PSA, prostate specific antigen. | | | | |

| **Table S5.** Baseline characteristics of participants according to fourths of waist at recruitment in men from UK Biobank. | | | | |
| --- | --- | --- | --- | --- |
|  | **Waist** | | | |
| **Characteristic** | **Q1** | **Q2** | **Q3** | **Q4** |
| No. of men | 56235 | 57565 | 50124 | 54285 |
|  |  |  |  |  |
| Sociodemographic |  |  |  |  |
| Age at recruitment (years), mean (SD) | 55.1 (8.5) | 56.4 (8.2) | 57.2 (8.0) | 57.7 (7.8) |
| Most deprived quintile, % (n) | 11779 (20.9) | 10543 (18.3) | 9583 (19.1) | 12892 (23.7) |
| No qualifications, % (n) | 7027 (12.5) | 7544 (13.1) | 7085 (14.1) | 7806 (14.4) |
| Black ethnicity, % (n) | 1077 (1.9) | 878 (1.5) | 667 (1.3) | 603 (1.1) |
| Not in paid/self-employment, % (n) | 19416 (34.5) | 21321 (37.0) | 19871 (39.6) | 23952 (44.1) |
| Living with partner, % (n) | 41574 (73.9) | 45266 (78.6) | 39374 (78.6) | 40149 (74.0) |
|  |  |  |  |  |
| Anthropometric |  |  |  |  |
| Height (cm), mean (SD) | 174.4 (6.8) | 175.4 (6.7) | 176.1 (6.8) | 176.7 (6.9) |
| BMI (kg/m^2^), mean (SD) | 23.8 (2.1) | 26.4 (1.9) | 28.5 (2.1) | 32.9 (4.0) |
| Body fat (%), mean (SD) | 19.6 (4.5) | 23.9 (3.6) | 26.7 (3.4) | 31.2 (4.1) |
| Waist circumference (cm), mean (SD) | 83.9 (4.5) | 93.1 (2.0) | 99.8 (2.0) | 111.9 (8.1) |
| Waist to hip ratio, mean (SD) | 0.869 (0.045) | 0.921 (0.036) | 0.954 (0.038) | 1.003 (0.052) |
|  |  |  |  |  |
| Lifestyle |  |  |  |  |
| Current cigarette smokers, % (n) | 7885 (14.0) | 6890 (12.0) | 6023 (12.0) | 6446 (11.9) |
| Drinking alcohol ≥ 20 g/day, % (n) | 21380 (38.0) | 25446 (44.2) | 23242 (46.4) | 24333 (44.8) |
| Physically inactive, % (n) | 11425 (20.3) | 14336 (24.9) | 14383 (28.7) | 20072 (37.0) |
|  |  |  |  |  |
| Health status |  |  |  |  |
| Vasectomy, % (n) | 2820 (5.0) | 3067 (5.3) | 2701 (5.4) | 2754 (5.1) |
| Hypertension, % (n) | 22688 (40.3) | 29307 (50.9) | 28342 (56.5) | 33520 (61.7) |
| Diabetes, % (n) | 1490 (2.6) | 2388 (4.1) | 3304 (6.6) | 7904 (14.6) |
|  |  |  |  |  |
| Prostate specific factors prior recruitment |  |  |  |  |
| PSA test, % (n) | 14499 (25.8) | 16166 (28.1) | 14535 (29.0) | 15236 (28.1) |
| Enlarged prostate, % (n) | 1619 (2.9) | 1950 (3.4) | 1708 (3.4) | 1795 (3.3) |
| Family history of prostate cancer, % (n) | 4152 (7.4) | 4292 (7.5) | 3874 (7.7) | 4063 (7.5) |
| ^1^ Values are means (SD).  Abbreviations: BMI, body mass index; PSA, prostate specific antigen. | | | | |

| **Table S6:** Mean and SD in men from UK Biobank with available imaging data (up to 4,800 men). | |
| --- | --- |
| **Imaging data** | **Mean (SD)** |
| MRI measures |  |
| Total adipose tissue volume, L | 19.64 (6.48) |
| Visceral adipose tissue, L | 4.94 (2.35) |
| Abdominal subcutaneous adipose tissue volume, L | 5.89 (2.53) |
| Muscle fat infiltration, % | 6.77 (1.72) |
| Liver proton density fat fraction, % | 4.71 (4.72) |
|  |  |
| DXA measures |  |
| Total tissue fat percentage, % | 30.29 (6.49) |
| Trunk fat mass, kg | 42.43 (7.91) |
| Trunk fat percentage, % | 35.53 (8.59) |
| Android fat mass, kg | 2.71 (1.25) |
| Android fat percentage, % | 0.42 (1.29) |
| Gynoid fat mass, kg | 3.60 (1.33) |
| Gynoid fat % | 0.32 (0.97) |
| Visceral adipose tissue mass, kg | 1.70 (0.95) |
| Visceral adipose tissue volume, cm^3^ | 1,798 (1,009) |

| **Table S7.** Pearson correlation coefficients between main adiposity measurements at baseline in 218,237 men from UK Biobank. | | | | |
| --- | --- | --- | --- | --- |
|  | **BMI** | **Body fat %** | **Waist circumference** | **Waist to hip ratio** |
| BMI | 1.000 |  |  |  |
| Body fat % | 0.799 | 1.000 |  |  |
| Waist circumference | 0.878 | 0.793 | 1.000 |  |
| Waist to hip ratio | 0.593 | 0.625 | 0.793 | 1.000 |
| All P values were < 0.001 | | | | |

| **Table S8.** Pearson correlation coefficients between adiposity measurements (**imaging visit**) with MRI adiposity measurements from the imagining in up to 11,501 men from UK Biobank. | | | | | | | | | |
| --- | --- | --- | --- | --- | --- | --- | --- | --- | --- |
| **MRI measurements** | **BMI** | **Body fat percentage** | **Waist** | **WHR** | **Total adipose tissue volume** | **Visceral adipose tissue** | **Abdominal subcutaneous adipose tissue volume** | **Muscle mass infiltration** | **Liver proton density fat fraction** |
| BMI | 1.000 |  |  |  |  |  |  |  |  |
| Body fat percentage | 0.777 | 1.000 |  |  |  |  |  |  |  |
| Waist | 0.861 | 0.773 | 1.000 |  |  |  |  |  |  |
| WHR | 0.574 | 0.588 | 0.777 | 1.000 |  |  |  |  |  |
| Total adipose tissue volume | 0.876 | 0.841 | 0.886 | 0.604 | 1.000 |  |  |  |  |
| Visceral adipose tissue | 0.784 | 0.767 | 0.804 | 0.672 | 0.853 | 1.000 |  |  |  |
| Abdominal subcutaneous adipose tissue volume | 0.853 | 0.790 | 0.829 | 0.541 | 0.940 | 0.693 | 1.000 |  |  |
| Muscle mass infiltration | 0.453 | 0.543 | 0.492 | 0.405 | 0.553 | 0.513 | 0.435 | 1.000 |  |
| Liver proton density fat fraction | 0.430 | 0.414 | 0.412 | 0.363 | 0.430 | 0.526 | 0.358 | 0.211 | 1.000 |
| All P values were < 0.001  Abbreviations: BMI, body mass index; MRI, magnetic resonance imaging; WHR, waist to hip ratio. | | | | | | | | | |

| **Table S9.** Pearson correlation coefficients between adiposity measurements (imaging visit) with DXA adiposity measurements from the imagining in up to 18,827 men from UK Biobank. | | | | | | | | |
| --- | --- | --- | --- | --- | --- | --- | --- | --- |
| **DXA measurements** | **BMI** | **Body fat percentage** | **Waist** | **WHR** | **Trunk fat mass** | **Android fat mass** | **Gynoid fat mass** | **VAT mass** |
| BMI | 1.000 |  |  |  |  |  |  |  |
| Body fat percentage | 0.777 | 1.000 |  |  |  |  |  |  |
| Waist | 0.861 | 0.773 | 1.000 |  |  |  |  |  |
| WHR | 0.574 | 0.588 | 0.777 | 1.000 |  |  |  |  |
| Trunk fat mass | 0.869 | 0.699 | 0.855 | 0.564 | 1.000 |  |  |  |
| Android fat mass | 0.889 | 0.834 | 0.889 | 0.660 | 0.801 | 1.000 |  |  |
| Gynoid fat mass | 0.853 | 0.768 | 0.846 | 0.496 | 0.739 | 0.878 | 1.000 |  |
| VAT mass | 0.809 | 0.761 | 0.808 | 0.658 | 0.755 | 0.936 | 0.738 | 1.000 |
| All P values were < 0.001  Abbreviations: BMI, body mass index; DXA, Dual-energy X-ray absorptiometry; VAT, visceral adipose tissue; WHR, waist to hip ratio. | | | | | | | | |

| **Table S10**. Mean difference in MRI- and DXA-derived body fat compartments per 1 SD higher levels of BMI, body fat percentage, waist circumference, and waist to hip ratio in men from UK Biobank. | | | | |
| --- | --- | --- | --- | --- |
|  | **Mean difference per 1 SD increase (95% CI)** | | | |
| **Adiposity measure** | **BMI** | **Body fat %** | **Waist circumference** | **WHR** |
| **MRI (max n=** **11,501)** |  |  |  |  |
| Total adipose tissue volume, L | 6.28 (6.18-6.38) | 5.95 (5.85-6.06) | 6.63 (6.52-6.75) | 4.68 (4.48-4.87) |
| Visceral adipose tissue, L | 1.89 (1.87-1.92) | 1.85 (1.82-1.88) | 1.94 (1.92-1.97) | 1.67 (1.64-1.70) |
| Abdominal subcutaneous adipose tissue volume, L | 2.22 (2.20-2.25) | 2.11 (2.08-2.14) | 2.17 (2.15-2.20) | 1.49 (1.45-1.53) |
| Muscle fat infiltration, % | 0.81 (0.79-0.84) | 0.88 (0.86-0.91) | 0.86 (0.83-0.88) | 0.67 (0.64-0.70) |
| Liver proton density fat fraction, % | 2.09 (1.90-2.28) | 2.10 (1.90-2.29) | 2.24 (2.03-2.44) | 2.06 (1.84-2.28) |
|  |  |  |  |  |
| **DXA (max n=18,827)** |  |  |  |  |
| Total tissue fat, % | 0.07 (0.06-0.09) | 0.08 (0.06-0.09) | 0.06 (0.04-0.07) | 0.03 (0.01-0.04) |
| Trunk fat mass, kg | 7.03 (6.99-7.07) | 5.88 (5.81-5.95) | 6.59 (6.54-6.64) | 4.70 (4.61-4.78) |
| Trunk fat, % | 0.09 (0.07-0.11) | 0.10 (0.08-0.12) | 0.08 (0.06-0.09) | 0.04 (0.02-0.06) |
| Android fat mass, kg | 1.11 (1.09-1.13) | 1.11 (1.08-1.14) | 1.20 (1.17-1.22) | 0.98 (0.94-1.02) |
| Android fat, % | 0.10 (0.08-0.12) | 0.11 (0.09-0.13) | 0.09 (0.07-0.11) | 0.05 (0.03-0.07) |
| Gynoid fat mass, kg | 1.13 (1.10-1.15) | 1.10 (1.07-1.13) | 1.18 (1.15-1.21) | 0.79 (0.73-0.84) |
| Gynoid fat, % | 0.063 (0.049-0.077) | 0.067 (0.052-0.081) | 0.050 (0.036-0.064) | 0.016 (0.001-0.030) |
| VAT mass, kg | 0.81 (0.79-0.83) | 0.77 (0.74-0.80) | 0.86 (0.84-0.89) | 0.74 (0.71-0.78) |
| VAT volume, cm^3^ | 858 (833-882) | 817 (790-845) | 914 (887-941) | 789 (752-825) |
| Multivariable linear regression model adjusted by age and height.  Abbreviations: BMI, body mass index; DXA, dual-energy x-ray absorptiometry; MRI, magnetic resonance imaging; VAT, visceral adipose tissue; WHR, waist to hip ratio. | | | | |

| **Table S11**. Geometric means of selected MRI measurements by tenths of anthropometric measurements at the imaging visit in up to 11,501 men from UK Biobank. | | | | | | | | | | | |
| --- | --- | --- | --- | --- | --- | --- | --- | --- | --- | --- | --- |
| **Anthropometric measurements at imaging visit** | | **Total adipose tissue volume, L** | | **Visceral adipose tissue, L** | | **Abdominal subcutaneous adipose tissue volume, L** | | **Muscle mass infiltration, %** | | **Liver proton density fat fraction, %** | |
|  | **Tenths** | **n** | **Mean (min-max)** | **n** | **Mean (min-max)** | **n** | **Mean (min-max)** | **n** | **Mean (min-max)** | **n** | **Mean (min-max)** |
| **BMI** | 1 | 379 | 11.23 (10.93-11.53) | 1152 | 2.08 (1.99-2.16) | 1152 | 3.04 (2.96-3.12) | 1131 | 5.66 (5.58-5.75) | 199 | 1.54 (0.95-2.14) |
|  | 2 | 402 | 13.91 (13.62-14.20) | 1205 | 2.98 (2.90-3.06) | 1205 | 3.95 (3.87-4.03) | 1189 | 5.97 (5.89-6.05) | 212 | 2.40 (1.82-2.97) |
|  | 3 | 367 | 15.73 (15.42-16.04) | 1147 | 3.59 (3.51-3.67) | 1147 | 4.46 (4.38-4.55) | 1133 | 6.18 (6.10-6.26) | 197 | 2.85 (2.25-3.45) |
|  | 4 | 395 | 16.90 (16.60-17.20) | 1156 | 4.03 (3.94-4.11) | 1157 | 4.85 (4.77-4.93) | 1136 | 6.38 (6.30-6.46) | 213 | 3.34 (2.76-3.91) |
|  | 5 | 379 | 18.42 (18.12-18.72) | 1133 | 4.61 (4.52-4.69) | 1133 | 5.30 (5.22-5.38) | 1114 | 6.60 (6.51-6.68) | 215 | 3.71 (3.14-4.28) |
|  | 6 | 407 | 19.70 (19.41-20.00) | 1125 | 5.06 (4.97-5.14) | 1125 | 5.74 (5.66-5.82) | 1105 | 6.68 (6.60-6.77) | 223 | 4.63 (4.07-5.19) |
|  | 7 | 383 | 21.54 (21.24-21.85) | 1141 | 5.63 (5.54-5.71) | 1141 | 6.33 (6.25-6.42) | 1114 | 7.03 (6.95-7.11) | 226 | 4.83 (4.27-5.39) |
|  | 8 | 372 | 23.26 (22.95-23.57) | 1096 | 6.06 (5.97-6.14) | 1096 | 6.92 (6.83-7.00) | 1077 | 7.27 (7.18-7.35) | 207 | 6.20 (5.62-6.78) |
|  | 9 | 357 | 26.12 (25.81-26.43) | 1157 | 6.93 (6.84-7.01) | 1157 | 7.82 (7.74-7.90) | 1129 | 7.54 (7.46-7.62) | 206 | 5.97 (5.38-6.55) |
|  | 10 | 318 | 32.19 (31.85-32.52) | 1168 | 8.57 (8.48-8.65) | 1163 | 10.62 (10.54-10.70) | 1119 | 8.40 (8.32-8.48) | 199 | 9.13 (8.54-9.73) |
| **Body fat percentage** |  |  |  |  |  |  |  |  |  |  |  |
|  | 1 | 375 | 10.88 (10.55-11.21) | 1142 | 1.99 (1.90-2.07) | 1142 | 2.91 (2.82-2.99) | 1118 | 5.53 (5.45-5.61) | 192 | 1.47 (0.86-2.08) |
|  | 2 | 379 | 14.02 (13.70-14.35) | 1134 | 2.97 (2.89-3.06) | 1134 | 3.92 (3.83-4.00) | 1108 | 5.90 (5.82-5.97) | 215 | 2.20 (1.63-2.78) |
|  | 3 | 416 | 16.09 (15.78-16.40) | 1211 | 3.72 (3.64-3.81) | 1212 | 4.56 (4.47-4.64) | 1189 | 6.16 (6.08-6.24) | 213 | 2.91 (2.34-3.49) |
|  | 4 | 383 | 16.94 (16.61-17.26) | 1154 | 4.13 (4.04-4.22) | 1154 | 4.96 (4.88-5.05) | 1140 | 6.37 (6.29-6.44) | 199 | 2.97 (2.38-3.57) |
|  | 5 | 415 | 18.74 (18.43-19.05) | 1142 | 4.74 (4.66-4.83) | 1142 | 5.45 (5.37-5.54) | 1126 | 6.49 (6.41-6.56) | 237 | 4.00 (3.46-4.54) |
|  | 6 | 356 | 20.38 (20.04-20.71) | 1041 | 5.14 (5.04-5.23) | 1041 | 5.87 (5.78-5.96) | 1022 | 6.80 (6.72-6.89) | 195 | 4.24 (3.64-4.84) |
|  | 7 | 343 | 21.17 (20.83-21.51) | 1120 | 5.62 (5.53-5.71) | 1120 | 6.30 (6.21-6.38) | 1100 | 6.89 (6.81-6.97) | 199 | 5.48 (4.88-6.07) |
|  | 8 | 392 | 23.56 (23.24-23.88) | 1126 | 6.20 (6.12-6.29) | 1126 | 7.01 (6.93-7.10) | 1103 | 7.25 (7.17-7.33) | 214 | 6.12 (5.55-6.69) |
|  | 9 | 353 | 26.09 (25.75-26.43) | 1099 | 6.95 (6.86-7.04) | 1098 | 7.91 (7.82-8.00) | 1069 | 7.75 (7.67-7.83) | 219 | 6.59 (6.03-7.16) |
|  | 10 | 299 | 31.87 (31.50-32.24) | 1058 | 8.40 (8.31-8.49) | 1054 | 10.62 (10.53-10.71) | 1023 | 8.70 (8.61-8.78) | 187 | 8.55 (7.93-9.16) |
| All models are adjusted for sex and height.  Abbreviations: BMI, body mass index; WHR, waist to hip ratio. | | | | | | | | | | | |
| **Table S11**. Continued | | | | | | | | | | | |
| **Anthropometric measurements at imaging visit** | | **Total adipose tissue volume, L** | | **Visceral adipose tissue, L** | | **Abdominal subcutaneous adipose tissue volume, L** | | **Muscle mass infiltration, %** | | **Liver proton density fat fraction, %** | |
|  | **Tenths** | **n** | **Mean (min-max)** | **n** | **Mean (min-max)** | **n** | **Mean (min-max)** | **n** | **Mean (min-max)** | **n** | **Mean (min-max)** |
| **Waist circumference** |  |  |  |  |  |  |  |  |  |  |  |
|  | 1 | 476 | 11.66 (11.38-11.94) | 1344 | 2.16 (2.08-2.24) | 1343 | 3.16 (3.08-3.24) | 1325 | 5.64 (5.56-5.71) | 251 | 1.64 (1.11-2.18) |
|  | 2 | 459 | 14.58 (14.30-14.87) | 1276 | 3.08 (3.00-3.16) | 1276 | 4.09 (4.01-4.18) | 1259 | 5.96 (5.88-6.03) | 240 | 2.42 (1.88-2.96) |
|  | 3 | 329 | 16.30 (15.97-16.64) | 834 | 3.65 (3.55-3.74) | 835 | 4.54 (4.44-4.64) | 828 | 6.28 (6.18-6.37) | 173 | 2.90 (2.27-3.54) |
|  | 4 | 512 | 17.49 (17.22-17.76) | 1407 | 4.08 (4.01-4.16) | 1408 | 4.95 (4.87-5.03) | 1387 | 6.39 (6.32-6.46) | 295 | 3.27 (2.78-3.76) |
|  | 5 | 340 | 19.05 (18.72-19.38) | 990 | 4.57 (4.48-4.65) | 990 | 5.44 (5.34-5.53) | 967 | 6.59 (6.51-6.68) | 176 | 4.57 (3.94-5.20) |
|  | 6 | 462 | 20.87 (20.59-21.16) | 1326 | 5.12 (5.04-5.19) | 1326 | 5.83 (5.75-5.91) | 1304 | 6.79 (6.72-6.87) | 263 | 4.94 (4.42-5.46) |
|  | 7 | 386 | 22.35 (22.04-22.66) | 1147 | 5.67 (5.59-5.76) | 1147 | 6.30 (6.21-6.38) | 1132 | 7.05 (6.97-7.13) | 205 | 5.61 (5.02-6.20) |
|  | 8 | 330 | 24.76 (24.42-25.09) | 1157 | 6.36 (6.27-6.44) | 1157 | 7.06 (6.97-7.14) | 1127 | 7.23 (7.15-7.31) | 208 | 6.13 (5.55-6.71) |
|  | 9 | 244 | 28.20 (27.81-28.59) | 940 | 7.11 (7.01-7.20) | 940 | 8.08 (7.98-8.17) | 910 | 7.66 (7.57-7.75) | 137 | 6.82 (6.10-7.54) |
|  | 10 | 220 | 33.56 (33.14-33.97) | 1084 | 8.68 (8.59-8.76) | 1079 | 10.66 (10.57-10.75) | 1033 | 8.59 (8.50-8.67) | 149 | 9.51 (8.82-10.19) |
| **WHR** |  |  |  |  |  |  |  |  |  |  |  |
|  | 1 | 449 | 13.36 (12.89-13.83) | 1204 | 2.44 (2.34-2.54) | 1203 | 3.69 (3.57-3.81) | 1176 | 5.80 (5.72-5.89) | 250 | 1.96 (1.41-2.51) |
|  | 2 | 513 | 16.16 (15.72-16.60) | 1316 | 3.40 (3.31-3.50) | 1317 | 4.55 (4.44-4.67) | 1294 | 6.19 (6.11-6.27) | 289 | 2.76 (2.25-3.27) |
|  | 3 | 499 | 17.32 (16.88-17.76) | 1244 | 3.87 (3.77-3.97) | 1244 | 4.98 (4.86-5.10) | 1226 | 6.31 (6.23-6.39) | 290 | 3.37 (2.87-3.88) |
|  | 4 | 495 | 18.63 (18.18-19.07) | 1333 | 4.30 (4.20-4.39) | 1333 | 5.30 (5.18-5.41) | 1307 | 6.47 (6.39-6.55) | 258 | 3.99 (3.46-4.53) |
|  | 5 | 419 | 20.61 (20.13-21.09) | 1208 | 4.90 (4.80-5.00) | 1208 | 5.88 (5.76-6.00) | 1175 | 6.73 (6.65-6.81) | 230 | 4.56 (3.99-5.13) |
|  | 6 | 394 | 20.81 (20.32-21.31) | 1173 | 5.11 (5.01-5.21) | 1173 | 5.94 (5.82-6.06) | 1154 | 6.76 (6.68-6.85) | 223 | 4.92 (4.34-5.50) |
|  | 7 | 360 | 23.39 (22.87-23.91) | 1100 | 5.79 (5.69-5.89) | 1100 | 6.70 (6.58-6.83) | 1072 | 7.04 (6.96-7.13) | 203 | 6.52 (5.91-7.13) |
|  | 8 | 258 | 24.16 (23.54-24.77) | 1042 | 6.24 (6.13-6.34) | 1042 | 6.91 (6.78-7.04) | 1026 | 7.33 (7.24-7.42) | 134 | 6.51 (5.76-7.26) |
|  | 9 | 237 | 26.89 (26.25-27.54) | 1008 | 6.96 (6.85-7.07) | 1009 | 7.90 (7.77-8.03) | 982 | 7.62 (7.52-7.71) | 136 | 7.34 (6.59-8.08) |
|  | 10 | 134 | 27.81 (26.95-28.66) | 877 | 8.11 (8.00-8.23) | 872 | 8.63 (8.49-8.77) | 860 | 8.08 (7.98-8.18) | 84 | 8.38 (7.44-9.32) |
| All models are adjusted for sex and height.  Abbreviations: BMI, body mass index; WHR, waist to hip ratio. | | | | | | | | | | | |

| **Table S12**. Geometric means of selected DXA measurements by tenths of anthropometric measurements **at the imaging visit** in up to 18,827 men from UK Biobank. | | | | | | | | | |
| --- | --- | --- | --- | --- | --- | --- | --- | --- | --- |
| **Anthropometric measurements at imaging visit** | | **Trunk fat mass, kg** | | **Android fat mass, kg** | | **Gynoid fat mass, kg** | | **VAT mass, kg** | |
|  | **Tenths** | **n** | **Mean (min-max)** | **n** | **Mean (min-max)** | **n** | **Mean (min-max)** | **n** | **Mean (min-max)** |
| **BMI** |  |  |  |  |  |  |  |  |  |
|  | 1 | 1958 | 32.3 (32.2-32.4) | 210 | 1.07 (0.98-1.16) | 210 | 2.13 (2.02-2.23) | 209 | 0.55 (0.47-0.62) |
|  | 2 | 1968 | 35.9 (35.7-36.0) | 239 | 1.61 (1.53-1.69) | 239 | 2.54 (2.44-2.64) | 238 | 0.88 (0.80-0.95) |
|  | 3 | 1818 | 37.7 (37.5-37.8) | 212 | 1.93 (1.84-2.01) | 212 | 2.88 (2.78-2.98) | 211 | 1.10 (1.03-1.18) |
|  | 4 | 1933 | 39.3 (39.2-39.5) | 224 | 2.14 (2.06-2.22) | 224 | 3.04 (2.94-3.14) | 223 | 1.25 (1.18-1.33) |
|  | 5 | 1930 | 40.8 (40.7-41.0) | 239 | 2.41 (2.33-2.49) | 239 | 3.26 (3.17-3.36) | 238 | 1.47 (1.40-1.54) |
|  | 6 | 1858 | 42.6 (42.4-42.7) | 248 | 2.68 (2.60-2.76) | 248 | 3.46 (3.37-3.55) | 245 | 1.71 (1.64-1.78) |
|  | 7 | 1837 | 44.2 (44.1-44.4) | 261 | 2.94 (2.86-3.01) | 261 | 3.76 (3.67-3.85) | 258 | 1.89 (1.82-1.96) |
|  | 8 | 1753 | 46.3 (46.2-46.5) | 246 | 3.25 (3.17-3.33) | 246 | 4.07 (3.97-4.16) | 245 | 2.13 (2.06-2.20) |
|  | 9 | 1838 | 49.3 (49.1-49.4) | 245 | 3.65 (3.57-3.73) | 245 | 4.50 (4.41-4.59) | 244 | 2.43 (2.36-2.50) |
|  | 10 | 1900 | 56.9 (56.8-57.1) | 235 | 5.04 (4.96-5.12) | 235 | 6.02 (5.93-6.12) | 223 | 3.34 (3.27-3.41) |
| **Body fat %** |  |  |  |  |  |  |  |  |  |
|  | 1 | 1899 | 33.8 (33.6-34.0) | 203 | 1.00 (0.91-1.09) | 203 | 2.05 (1.95-2.16) | 201 | 0.52 (0.44-0.60) |
|  | 2 | 1877 | 36.7 (36.5-36.9) | 225 | 1.57 (1.49-1.66) | 225 | 2.53 (2.43-2.63) | 223 | 0.87 (0.79-0.95) |
|  | 3 | 1958 | 38.5 (38.3-38.8) | 246 | 2.00 (1.91-2.08) | 246 | 2.88 (2.78-2.98) | 244 | 1.17 (1.10-1.25) |
|  | 4 | 1828 | 39.8 (39.5-40.0) | 218 | 2.15 (2.06-2.24) | 218 | 3.06 (2.96-3.17) | 217 | 1.28 (1.20-1.36) |
|  | 5 | 1896 | 41.2 (41.0-41.4) | 255 | 2.44 (2.36-2.52) | 255 | 3.29 (3.19-3.38) | 255 | 1.50 (1.42-1.57) |
|  | 6 | 1719 | 42.6 (42.4-42.8) | 226 | 2.78 (2.69-2.87) | 226 | 3.61 (3.51-3.71) | 226 | 1.75 (1.67-1.83) |
|  | 7 | 1852 | 43.8 (43.6-44.0) | 232 | 2.94 (2.86-3.03) | 232 | 3.74 (3.64-3.84) | 229 | 1.87 (1.79-1.95) |
|  | 8 | 1811 | 45.8 (45.6-46.0) | 251 | 3.34 (3.26-3.42) | 251 | 4.09 (4.00-4.19) | 249 | 2.22 (2.14-2.29) |
|  | 9 | 1789 | 48.3 (48.1-48.6) | 254 | 3.69 (3.61-3.77) | 254 | 4.53 (4.44-4.63) | 251 | 2.43 (2.36-2.51) |
|  | 10 | 1785 | 55.1 (54.9-55.3) | 219 | 4.93 (4.85-5.02) | 219 | 6.00 (5.89-6.10) | 209 | 3.19 (3.11-3.27) |

| **Table S12**. Continued. | | | | | | | | | | | | | | | | |
| --- | --- | --- | --- | --- | --- | --- | --- | --- | --- | --- | --- | --- | --- | --- | --- | --- |
| **Anthropometric measurements at imaging visit** | | | | **Trunk fat mass, kg** | | | **Android fat mass, kg** | | | | **Gynoid fat mass, kg** | | | | **VAT mass, kg** | |
|  | **Tenths** | | | **n** | **Mean (min-max)** | | **n** | | **Mean (min-max)** | | **n** | | **Mean (min-max)** | | **n** | **Mean (min-max)** |
|  | |  |  | | |  | |  | |  | |  | |  | | |
| **Waist circumference** |  | | |  |  | |  | |  | |  | |  | |  |  |
|  | 1 | | | 2294 | 33.6 (33.4-33.8) | | 268 | | 1.16 (1.08-1.23) | | 268 | | 2.21 (2.12-2.30) | | 266 | 0.60 (0.53-0.66) |
|  | 2 | | | 2097 | 36.6 (36.4-36.7) | | 253 | | 1.71 (1.63-1.79) | | 253 | | 2.65 (2.56-2.75) | | 252 | 0.96 (0.89-1.03) |
|  | 3 | | | 1372 | 38.2 (38.0-38.4) | | 199 | | 1.99 (1.90-2.08) | | 199 | | 2.94 (2.83-3.05) | | 198 | 1.16 (1.08-1.24) |
|  | 4 | | | 2234 | 39.6 (39.4-39.8) | | 323 | | 2.26 (2.19-2.33) | | 323 | | 3.17 (3.08-3.25) | | 321 | 1.35 (1.28-1.41) |
|  | 5 | | | 1569 | 40.9 (40.7-41.1) | | 208 | | 2.50 (2.41-2.58) | | 208 | | 3.37 (3.26-3.47) | | 206 | 1.53 (1.45-1.60) |
|  | 6 | | | 2119 | 42.5 (42.3-42.7) | | 305 | | 2.81 (2.74-2.88) | | 305 | | 3.59 (3.50-3.68) | | 304 | 1.82 (1.76-1.89) |
|  | 7 | | | 1899 | 44.3 (44.1-44.5) | | 229 | | 3.14 (3.05-3.22) | | 229 | | 3.89 (3.79-3.99) | | 227 | 2.06 (1.98-2.13) |
|  | 8 | | | 1924 | 46.4 (46.2-46.6) | | 225 | | 3.52 (3.44-3.61) | | 225 | | 4.30 (4.20-4.40) | | 224 | 2.32 (2.24-2.39) |
|  | 9 | | | 1528 | 49.4 (49.2-49.6) | | 177 | | 4.04 (3.94-4.13) | | 177 | | 4.92 (4.81-5.04) | | 175 | 2.71 (2.62-2.79) |
|  | 10 | | | 1791 | 56.5 (56.3-56.7) | | 173 | | 5.31 (5.22-5.41) | | 173 | | 6.29 (6.17-6.41) | | 162 | 3.48 (3.39-3.57) |
| **WHR** |  | | |  |  | |  | |  | |  | |  | |  |  |
|  | 1 | | | 1996 | 35.2 (35.0-35.5) | | 266 | | 1.43 (1.32-1.54) | | 266 | | 2.58 (2.45-2.71) | | 264 | 0.77 (0.68-0.85) |
|  | 2 | | | 1967 | 37.9 (37.6-38.2) | | 310 | | 1.93 (1.83-2.03) | | 310 | | 2.99 (2.86-3.11) | | 309 | 1.09 (1.01-1.17) |
|  | 3 | | | 1904 | 39.2 (39.0-39.5) | | 318 | | 2.29 (2.18-2.39) | | 318 | | 3.23 (3.11-3.36) | | 316 | 1.35 (1.27-1.42) |
|  | 4 | | | 2011 | 40.3 (40.1-40.6) | | 305 | | 2.51 (2.40-2.61) | | 305 | | 3.40 (3.27-3.52) | | 305 | 1.54 (1.46-1.62) |
|  | 5 | | | 1862 | 41.9 (41.6-42.1) | | 267 | | 2.76 (2.65-2.87) | | 267 | | 3.66 (3.53-3.80) | | 265 | 1.74 (1.65-1.82) |
|  | 6 | | | 1902 | 42.3 (42.1-42.6) | | 248 | | 2.97 (2.85-3.09) | | 248 | | 3.79 (3.65-3.93) | | 247 | 1.90 (1.81-1.99) |
|  | 7 | | | 1822 | 44.8 (44.5-45.0) | | 234 | | 3.44 (3.32-3.56) | | 234 | | 4.19 (4.05-4.33) | | 231 | 2.29 (2.19-2.38) |
|  | 8 | | | 1808 | 45.3 (45.0-45.5) | | 162 | | 3.48 (3.34-3.62) | | 162 | | 4.19 (4.02-4.36) | | 160 | 2.29 (2.18-2.40) |
|  | 9 | | | 1844 | 48.1 (47.8-48.3) | | 153 | | 4.08 (3.94-4.23) | | 153 | | 4.75 (4.57-4.93) | | 147 | 2.78 (2.67-2.90) |
|  | 10 | | | 1711 | 51.4 (51.1-51.7) | | 97 | | 4.66 (4.48-4.85) | | 97 | | 5.19 (4.97-5.41) | | 91 | 3.17 (3.02-3.32) |
| All models are adjusted for sex and height.  Abbreviations: BMI, body mass index; WHR, waist to hip ratio. | | | | | | | | | | | | | | | | |

| **Table S13.** Minimally- and multivariable-adjusted hazard ratios (95% CI) for prostate cancer death in relation to adiposity measurements at baseline in men from UK Biobank. | | | | | |
| --- | --- | --- | --- | --- | --- |
| **Anthropometric** | **Total n** | **PCa deaths** | **HR (95% CI) Minimally-adjusted** | **HR (95% CI) Multivariable-adjusted** | **Final model:**  **HR (95% CI) Multivariable-adjusted + PSA** |
| BMI, kg/m^2^ |  |  |  |  |  |
| Q1, <=25.0 | 55669 | 148 | 1 ref | 1 ref | 1 ref |
| Q2, 25.1 - 27.2 | 54197 | 160 | 1.03 (0.83 - 1.29) | 1.04 (0.83 - 1.30) | 1.04 (0.83 - 1.31) |
| Q3, 27.3-30.0 | 54695 | 191 | 1.22 (0.99 - 1.51) | 1.21 (0.98 - 1.51) | 1.21 (0.98 - 1.51) |
| Q4, >=30.1 | 53286 | 160 | 1.10 (0.88 - 1.38) | 1.07 (0.85 - 1.34) | 1.06 (0.84 - 1.34) |
| Per 5 kg/m^2^ increase | 217832 | 659 | 1.09 (0.99 - 1.19) | 1.07 (0.97 - 1.18) | 1.07 (0.97 - 1.17) |
| Body fat, % |  |  |  |  |  |
| Q1, <=21.5 | 56238 | 139 | 1 ref | 1 ref | 1 ref |
| Q2, 21.6 - 25.4 | 57568 | 137 | 0.81 (0.64 - 1.02) | 0.82 (0.65 - 1.04) | 0.82 (0.65 - 1.04) |
| Q3, 25.5-29.1 | 50128 | 178 | 0.98 (0.78 - 1.22) | 0.99 (0.79 - 1.24) | 0.98 (0.78 - 1.23) |
| Q4, >=29.2 | 54291 | 191 | 0.96 (0.77 - 1.19) | 0.96 (0.76 - 1.21) | 0.95 (0.76 - 1.20) |
| Per 5 % increase | 214211 | 645 | 1.01 (0.94 - 1.08) | 1.01 (0.94 - 1.08) | 1.00 (0.94 - 1.08) |
| Waist circumference, cm |  |  |  |  |  |
| Q1, <=89 | 56238 | 124 | 1 ref | 1 ref | 1 ref |
| Q2, 89.1 - 96.0 | 57568 | 165 | 1.14 (0.90 - 1.44) | 1.11 (0.88 - 1.40) | 1.10 (0.87 - 1.40) |
| Q3, 96.1-103.0 | 50128 | 165 | 1.23 (0.97 - 1.55) | 1.15 (0.91 - 1.46) | 1.15 (0.91 - 1.46) |
| Q4, >=103.1 | 54291 | 205 | 1.40 (1.12 - 1.75) | 1.25 (0.99 - 1.58) | 1.24 (0.98 - 1.57) |
| Per 10 cm increase | 218209 | 659 | 1.11 (1.03 - 1.18) | 1.06 (0.99 - 1.14) | 1.06 (0.99 - 1.14) |
| Waist to hip ratio |  |  |  |  |  |
| Q1, <=0.892 | 54636 | 116 | 1 ref | 1 ref | 1 ref |
| Q2, 0.893 - 0.934 | 54486 | 139 | 1.02 (0.80 - 1.31) | 1.00 (0.78 - 1.28) | 1.00 (0.78 - 1.28) |
| Q3, 0.935-0.978 | 54568 | 190 | 1.27 (1.01 - 1.60) | 1.22 (0.97 - 1.55) | 1.22 (0.96 - 1.54) |
| Q4, >=0.979 | 54483 | 214 | 1.34 (1.07 - 1.68) | 1.26 (1.00 - 1.59) | 1.25 (0.99 - 1.58) |
| Per 0.05 increase | 218157 | 659 | 1.09 (1.03 - 1.16) | 1.07 (1.01 - 1.14) | 1.07 (1.01 - 1.14) |
| Cox regression analyses.  Minimally-adjusted models are stratified by region and age at recruitment and adjusted for age (underlying time variable).  Multivariable-adjusted models are stratified by region and age at recruitment and adjusted for age (underlying time variable), Townsend deprivation score, ethnicity, lives with a wife or partner, smoking, physical activity, alcohol consumption, height, diabetes, and history of PSA test. Full details for each covariate are provided in the statistical section.  Abbreviations: BMI, body mass index; PCa, prostate cancer. | | | | | |

| **Table S14.** Multivariable-adjusted hazard ratios (95 % CI) for prostate cancer in relation to BMI, waist circumference and WHR using the WHO cut-off points at recruitment in men from UK Biobank. | | | | | | | | |
| --- | --- | --- | --- | --- | --- | --- | --- | --- |
|  | **BMI (kg/m^2^)** | | | **Waist circumference (cm)** | | | **WHR** | |
|  | **<25** | **25-29.9** | **≥30** | **<94** | **94-101.9** | **≥ 102 (higher risk)** | **<0.90** | **≥ 0.90** |
| N pca deaths | 144 | 342 | 173 | 220 | 191 | 248 | 137 | 522 |
| PCa death | 1 ref | 1.10 (0.90 - 1.34) | 1.08 (0.86 - 1.36) | 1 ref | 1.01 (0.83 - 1.23) | 1.16 (0.96 - 1.40) | 1 ref | 1.15 (0.95 - 1.40) |
| Cox regression analysis. Multivariable-adjusted models are stratified by region and age at recruitment and adjusted for age (underlying time variable), Townsend deprivation score, ethnicity, lives with a wife or partner, smoking, physical activity, alcohol consumption, height, diabetes, and history of PSA test. Full details for each covariate are provided in the statistical section.  Abbreviations: BMI, body mass index; PCa, prostate cancer; WHR, waist to hip ratio. | | | | | | | | |

# References

1. WCRF/AICR. World Cancer Research Fund International/American Institute for Cancer Research Continuous Update Project Report: Diet, Nutrition, Physical Activity, and Prostate Cancer. Available at: <http://www.wcrf.org/sites/default/files/Prostate-Cancer-SLR-2014.pdf>. 2014.

2. Genkinger JM, Wu K, Wang M, et al. Measures of body fatness and height in early and mid-to-late adulthood and prostate cancer: risk and mortality in The Pooling Project of Prospective Studies of Diet and Cancer. *Ann Oncol* 2020; **31**: 103-14.

3. Stroup DF, Berlin JA, Morton SC, et al. Meta-analysis of observational studies in epidemiology: a proposal for reporting. Meta-analysis Of Observational Studies in Epidemiology (MOOSE) group. *JAMA* 2000; **283**: 2008-12.

4. Ouzzani M, Hammady H, Fedorowicz Z, Elmagarmid A. Rayyan-a web and mobile app for systematic reviews. *Syst Rev* 2016; **5**: 210.

5. Gapstur SM, Gann PH, Colangelo LA, et al. Postload plasma glucose concentration and 27-year prostate cancer mortality (United States). *Cancer Causes Control* 2001; **12**: 763-72.

6. Rodriguez C, Patel AV, Calle EE, Jacobs EJ, Chao A, Thun MJ. Body mass index, height, and prostate cancer mortality in two large cohorts of adult men in the United States. *Cancer Epidemiol Biomarkers Prev* 2001; **10**: 345-53.

7. Calle EE, Rodriguez C, Walker-Thurmond K, Thun MJ. Overweight, obesity, and mortality from cancer in a prospectively studied cohort of U.S. adults. *N Engl J Med* 2003; **348**: 1625-38.

8. Eichholzer M, Bernasconi F, Jordan P, Stahelin HB. Body mass index and the risk of male cancer mortality of various sites: 17-year follow-up of the Basel cohort study. *Swiss Med Wkly* 2005; **135**: 27-33.

9. Fujino Y, Japan Collaborative Cohort Study for Evaluation of C. Anthropometry, development history and mortality in the Japan Collaborative Cohort Study for Evaluation of Cancer (JACC). *Asian Pac J Cancer Prev* 2007; **8 Suppl**: 105-12.

10. Martin RM, Vatten L, Gunnell D, Romundstad P, Nilsen TI. Components of the metabolic syndrome and risk of prostate cancer: the HUNT 2 cohort, Norway. *Cancer Causes Control* 2009; **20**: 1181-92.

11. Burton A, Martin R, Galobardes B, Davey Smith G, Jeffreys M. Young adulthood body mass index and risk of cancer in later adulthood: historical cohort study. *Cancer Causes Control* 2010; **21**: 2069-77.

12. Batty GD, Kivimaki M, Clarke R, Davey Smith G, Shipley MJ. Modifiable risk factors for prostate cancer mortality in London: forty years of follow-up in the Whitehall study. *Cancer Causes Control* 2011; **22**: 311-8.

13. Dehal A, Garrett T, Tedders SH, Arroyo C, Afriyie-Gyawu E, Zhang J. Body mass index and death rate of colorectal cancer among a national cohort of U.S. adults. *Nutr Cancer* 2011; **63**: 1218-25.

14. Discacciati A, Orsini N, Andersson SO, Andren O, Johansson JE, Wolk A. Body mass index in early and middle-late adulthood and risk of localised, advanced and fatal prostate cancer: a population-based prospective study. *Br J Cancer* 2011; **105**: 1061-8.

15. Gray L, Lee IM, Sesso HD, Batty GD. Association of body mass index in early adulthood and middle age with future site-specific cancer mortality: the Harvard Alumni Health Study. *Ann Oncol* 2012; **23**: 754-9.

16. Haggstrom C, Stocks T, Ulmert D, et al. Prospective study on metabolic factors and risk of prostate cancer. *Cancer* 2012; **118**: 6199-206.

17. Taghizadeh N, Boezen HM, Schouten JP, Schroder CP, Elisabeth de Vries EG, Vonk JM. BMI and lifetime changes in BMI and cancer mortality risk. *PLoS One* 2015; **10**: e0125261.

18. Hong JS, Yi SW, Yi JJ, Hong S, Ohrr H. Body Mass Index and Cancer Mortality Among Korean Older Middle-Aged Men: A Prospective Cohort Study. *Medicine* 2016; **95**.

19. Perez-Cornago A, Appleby PN, Pischon T, et al. Tall height and obesity are associated with an increased risk of aggressive prostate cancer: results from the EPIC cohort study. *BMC Med* 2017; **15**: 115.

20. Dickerman BA, Torfadottir JE, Valdimarsdottir UA, et al. Body fat distribution on computed tomography imaging and prostate cancer risk and mortality in the AGES-Reykjavik study. *Cancer* 2019; **125**: 2877-85.

21. Jochems SHJ, Stattin P, Haggstrom C, et al. Height, body mass index and prostate cancer risk and mortality by way of detection and cancer risk category. *Int J Cancer* 2020; **147**: 3328-38.

22. Jochems SHJ, Wood AM, Haggstrom C, Orho-Melander M, Stattin P, Stocks T. Waist circumference and a body shape index and prostate cancer risk and mortality. *Cancer Med* 2021; **10**: 2885-96.
